# Discrimination between *E*. *granulosus sensu stricto*, *E*. *multilocularis* and *E*. *shiquicus* Using a Multiplex PCR Assay

**DOI:** 10.1371/journal.pntd.0004084

**Published:** 2015-09-22

**Authors:** Cong-Nuan Liu, Zhong-Zi Lou, Li Li, Hong-Bin Yan, David Blair, Meng-Tong Lei, Jin-Zhong Cai, Yan-Lei Fan, Jian-Qiu Li, Bao-Quan Fu, Yu-Rong Yang, Donald P. McManus, Wan-Zhong Jia

**Affiliations:** 1 State Key Laboratory of Veterinary Etiological Biology/Key Laboratory of Veterinary Parasitology of Gansu Province/Key Laboratory of Zoonoses of Agriculture Ministry/Lanzhou Veterinary Research Institute, CAAS, Lanzhou, People's Republic of China; 2 School of Marine and Tropical Biology, James Cook University, Queensland, Australia; 3 Qinghai Academy of Animal Science and Veterinary Medicine, Xining, People's Republic of China; 4 Molecular Parasitology Laboratory, Infectious Diseases Division, QIMR Berghofer Medical Research Institute, Brisbane, Queensland, Australia; 5 Ningxia Medical University, Yinchuan, Ningxia Hui Autonomous Region, People's Republic of China; University of Melbourne, AUSTRALIA

## Abstract

**Background:**

Infections of *Echinococcus granulosus sensu stricto* (s.s), *E*. *multilocularis* and *E*. *shiquicus* are commonly found co-endemic on the Qinghai-Tibet plateau, China, and an efficient tool is needed to facilitate the detection of infected hosts and for species identification.

**Methodology/Principal Findings:**

A single-tube multiplex PCR assay was established to differentiate the *Echinococcus* species responsible for infections in intermediate and definitive hosts. Primers specific for *E*. *granulosus*, *E*. *multilocularis* and *E*. *shiquicus* were designed based on sequences of the mitochondrial NADH dehydrogenase subunit 1 (*nad*1), NADH dehydrogenase subunit 5 (*nad*5) and cytochrome c oxidase subunit 1 (*cox*1) genes, respectively. This multiplex PCR accurately detected *Echinococcus* DNA without generating nonspecific reaction products. PCR products were of the expected sizes of 219 (*nad*1), 584 (*nad*5) and 471 (*cox*1) bp. Furthermore, the multiplex PCR enabled diagnosis of multiple infections using DNA of protoscoleces and copro-DNA extracted from fecal samples of canine hosts. Specificity of the multiplex PCR was 100% when evaluated using DNA isolated from other cestodes. Sensitivity thresholds were determined for DNA from protoscoleces and from worm eggs, and were calculated as 20 pg of DNA for *E*. *granulosus* and *E*. *shiquicus*, 10 pg of DNA for *E*. *multilocularis*, 2 eggs for *E*. *granulosus*, and 1 egg for *E*. *multilocularis*. Positive results with copro-DNA could be obtained at day 17 and day 26 after experimental infection of dogs with larval *E*. *multilocularis* and *E*. *granulosus*, respectively.

**Conclusions/Significance:**

The multiplex PCR developed in this study is an efficient tool for discriminating *E*. *granulosus*, *E*. *multilocularis* and *E*. *shiquicus* from each other and from other taeniid cestodes. It can be used for the detection of canids infected with *E*. *granulosus s*.*s*. and *E*. *multilocularis* using feces collected from these definitive hosts. It can also be used for the identification of the *Echinococcus* metacestode larva in intermediate hosts, a stage that often cannot be identified to species on visual inspection.

## Author Summary

The canid adapted intestinal tapeworms, *Echinococcus granulosus*, *E*. *multilocularis* and *E*. *shuiqucus* are well known to be endemic in Northwestern China. The first two species can cause fatal disease in humans. Although *E*. *shiquicus* has not been reported to infect humans, all three species can be transmitted by dogs. The very close relationship between dogs and humans can readily lead to human infection. To aid the surveillance and management of echinococcosis, effective diagnostic approaches are urgently needed. We developed a single tube multiplex PCR assay for the accurate identification and discrimination of the three *Echinococcus* species for use in both clinical diagnosis and epidemiological studies.

## Introduction

In the most recent taxonomic revision, nine species were recognized in the genus *Echinococcus* [1]. Of these, the most important and widespread are *E*. *granulosus sensu stricto* (genotypes G1-G3) and *E*. *multilocularis*, which cause cystic echinococcosis (CE) and alveolar echinococcosis (AE), respectively. The former is commonly associated with livestock and human infections worldwide whereas the latter is primarily found in voles and humans and is geographically limited to the northern hemisphere [2]. To date, *E*. *granulosus s*.*s*., *E*. *canadensis* (G6), *E*. *multilocularis* and *E*. *shiquicus* have been identified in China [3–5]. Both *E*. *multilocularis* and *E*. *granulosus s*.*s*. are particularly widespread in western China, including Qinghai, Ningxia, Gansu, Xinjiang and Sichuan provinces/autonomous regions, and are well known as major public health and medical threats. Unlike the other species, *E*. *shiquicus* has a very restricted distribution, being reported only from Qinghai Province, China. This species is not known to cause human echinococcosis. The intermediate hosts are plateau pikas (*Ochotona curzoniae*), in which unilocular cysts occur.

For *Echinococcus* species in general, dogs, wolves, other canids and cats are definitive hosts in which adult worms cause sub-clinical infections [6–9]. However, larval *Echinococcus* spp. can cause morbidity and mortality in their intermediate hosts which include cattle, sheep, small mammals (including rodents, plateau pikas, etc.) and humans [10, 11]. It can be difficult to discriminate morphologically adults of some *Echinococcus* species, such as *E*. *multilocularis* and *E*. *shiquicus* [12].

To replace traditional morphological methods, a number of molecular approaches targeting parasite DNA have been developed for identification/discrimination of different life stages of *Echinococcus* species in definitive and intermediate hosts [13–15]. Multiplex PCR approaches, simultaneously using multiple specific primers in a single tube and detecting more than one target species, are material- and time-saving, precise, efficient and cost-effective when DNA from a mixture of pathogens may be present in a sample. This approach is also suitable for mass-screening of samples that may be generated from epidemiological investigations in endemic areas. Several multiplex PCR methods have been developed for identifying certain *Echinococcus* species, but none for the identification of *E*. *shiquicus* [16–17].

Based on interspecific variation in mitochondrial genes of the genus *Echinococcus*, we designed a multiplex PCR assay with three pairs of specific primers in a single reaction tube for rapid identification of *E*. *granulosus s*.*s*., *E*. *multilocularis* and *E*. *shiquicus* originating from either intermediate or definitive hosts. Further assessment of the sensitivity and specificity of the multiplex PCR assay was performed using metacestode DNA and copro-DNA to determine the reliability and accuracy of the new diagnostic tool developed in this study.

## Materials and Methods

### Ethics statement

Dogs and mice used in this study were handled in strict accordance with good animal practice according to the Animal Ethics Procedures and Guidelines of the People's Republic of China (Regulations for Administration of Affairs Concerning Experimental Animals, China, 1988). No endangered/protected species were involved in this study. The dogs and mice used were also treated in accordance with the institutional procedures and guidelines for animal husbandry issued by the Ethics Committee of Lanzhou Veterinary Research Institute, Chinese Academy of Agricultural Sciences (Approval No. LVRIEC2010-005).

### Sampling of *Echinococcus* material

Adult worms were collected from stray dogs during routine work of the endemic echinococcosis prevention and control program in Dari County, Qinghai Province, P.R. China. A total of 86 *Echinococcus* spp. metacestode samples from yaks, sheep, Qinghai voles (*Microtus/Neodon fuscus*) and plateau pikas were collected on the Qinghai-Tibet plateau, P.R. China. Ten yak lungs and 16 sheep livers harboring hydatid cysts were collected from abattoirs in Maqu County, Gansu Province and Xining City, Qinghai Province, respectively. Thirty Qinghai vole livers and 30 plateau pika lungs harboring hydatid cysts were provided by the epidemic prevention station of Dari County, Qinghai Province.

Parasite materials were dissected from the host tissue and stored either in 70% ethanol before molecular analyses, or temporarily stored at 4°C prior to experimental infections of dogs.

### Experimental infection of dogs

Fifteen dogs (mixed breeds) aged 6–8 months were purchased in Lanzhou City, Gansu Province, China. These were de-wormed using praziquantel and confirmed to be free of intestinal parasites by examination of their feces two weeks later. Samples of these feces were retained as negative controls for the multiplex PCR assay. Live protoscoleces (100,000) of each *Echinococcus* spp. were fed independently to five dogs after their viability for dog challenge was confirmed by microscopy.

### Sampling of adults/eggs of *Echinococcus* spp. from challenged dogs

Dogs were euthanized three months after challenge with protoscoleces. Fecal samples were collected from the dogs each day prior to sacrifice. After removal of the coarse gut contents, the small intestine was cut into 15–20 cm lengths and opened to expose the mucosa. Samples, taken by scraping the mucosa with glass strips, were placed in petri dishes in bio-safety containers [18]. After addition of a small volume of sterile phosphate-buffered saline (PBS, pH 7.2), the contents were checked for the presence of worms (intact or fragmented) and/or eggs. Adult worms were removed using a glass needle and washed in PBS three times. All procedures were performed following appropriate bio-safety conditions [19].

### Fecal sampling from non-experimented definitive hosts

Ten stray dogs, provided by the epidemic prevention station in Dari County, Qinghai Province, were processed as above to obtain mucosal samples, worms and eggs. Additionally, five fecal samples from captive foxes were collected from a fur farm in Lanzhou City, Gansu Province. All the collected fecal samples were frozen at -80°C for at least seven days for bio-safety reasons. Worm samples were preserved either in 70% ethanol or frozen at (-80°C) in PBS for further analyses.

### Other helminths

DNA samples, extracted from a variety of cestodes (identities confirmed by sequencing and morphology), were used to determine the specificity of the newly developed multiplex PCR assay (Table 1). They were kindly provided by the Key Laboratory of Veterinary Parasitology of Gansu Province, Lanzhou Veterinary Research Institute, CAAS.

**Table 1:** Parasite species and their GenBank accession numbers for the *nad*1, *nad*5 and *cox*1 genes used in this study.

Parasite	Sequence accession no.	Reference
*E*. *granulosus s*.*s*.	NC_008075/ AF297617	Yang *et al*, 2005 [22]; Le *et al*, 2000 [35].
*E*. *multilocularis*	NC_000928	Nakao *et al*, 2002 [23].
*E*. *shiquicus*	NC_009460	Nakao *et al*, 2007 [24].
*E*. *oligarthrus*	AB208545/ NC_009461	Nakao *et al*, 2007 [24].
*E*. *canadensis*	NC_011121/ AB235847	Nakao *et al*, 2007 [24].
*E*. *ortleppi*	NC_011122	Nakao *et al*, 2007 [24].
*E*. *vogeli*	NC_009462	Nakao *et al*, 2007 [24].
*E*. *canadensis* (G6)*	AB208063	Nakao *et al*, 2007 [24].
*T*. hydatigena*	GQ228819/ FJ518620	Jia *et al*, 2010 [36]; Liu *et al*, 2011 [37].
*T*. multiceps*	GQ228818/ FJ495086	Jia *et al*, 2010 [36]; Liu *et al*, 2011 [37].
*T*. pisiformis*	GU569096	Jia *et al*, 2010 [36].
*T*. *asiatica*	AF445798	Jeon *et al*, 2005 [38].
*T*. *saginata*	NC009938/ AY684274	Jeon *et al*, 2007 [39].
*T*. taeniaeformis*	JQ663994/ FJ597547	Jia *et al*. 2012 [40]; Liu *et al*, 2011 [37].
*T*. solium*	AB086256	Nakao *et al*, 2003 [41].
*D*. caninum*	AB732959	Nakao *et al*, 2013 [42].

Note: DNA from parasites labeled with * were used to test the specificity of the multiplex PCR system in this study.

### Host tissue sampling

DNA extracted from host tissues was used to check for nonspecific reactions or assay interference that might be caused by contamination of parasite samples with host DNA. Host tissues included dog intestines, and liver and lung samples from cattle, sheep, Qinghai voles and plateau pikas.

### DNA extraction from samples

Two hundred mg of each metacestode sample was frozen in liquid nitrogen and ground to powder after removal of ethanol or PBS by rinsing with ddH_2_O. Total genomic DNA was extracted using a QIAGEN DNeasy Blood & Tissue Kit (QIAGEN, Hilden, Germany) according to the manufacturer’s instructions and stored at -20°C until use.

To minimize the impact of inhibitors on PCR using copro-samples as template, an additional step of stool flotation in saturated zinc chloride solution was used before copro-DNA extraction [20]. Briefly, about 20 g (20 ml) fecal material was placed in a 50 ml centrifuge tube, which was then filled with zinc chloride solution. The tube was vortexed until the fecal material was completely broken up. The tube was then centrifuged at 1000 ×g for 5 min. Five hundred μl of the supernatant (usually containing helminth eggs, proglottids or cells of parasites) was transferred to a 2 ml centrifuge tube, 1.5 ml ddH_2_O was added to dilute the solution, and the tube was centrifuged at 12,000 ×g for 10 min. The supernatant was carefully discarded and 200 μl ddH_2_O added to suspend the sediment for DNA extraction. Total genomic DNA was extracted using a QIAGEN QIAamp DNA Stool Mini Kit (QIAGEN, Hilden, Germany) following the manufacturer’s instructions, and the DNA concentration was determined using a spectrophotometer (Thermo, NanoDrop 2000, USA) after elution in 50 μl ddH_2_O for use in the PCR assay.

Genomic DNA was extracted from host tissues using a QIAGEN DNeasy Blood & Tissue Kit (QIAGEN, Hilden, Germany), according to the manufacturer’s instructions, and stored at -20°C until use.

### Primer design

The complete mt genomes (mtDNA) of various cestodes (Table 1) available in GenBank (http://www.ncbi.nlm.nih.gov/) were retrieved to facilitate design of primers specific for *E*. *granulosus s*.*s*., *E*. *multilocularis* and *E*. *shiquicus* (Table 1). The sequences were aligned automatically using Clustal in MEGA5.0 [21]. Primer pairs, expected to be specific for *E*. *granulosus s*.*s*. (S1 Fig), *E*. *multilocularis* (S2 Fig) and *E*. *shiquicus* (S3 Fig), were thus obtained. After some preliminary experimentation, one pair of primers specific for each *Echinococcus* spp. was selected for inclusion in the multiplex PCR assay. Sequences of these primers, target genes and other related information are presented in Table 2.

**Table 2:** Sequences of primers used in the multiplex PCR.

Species	Gene	Primer	Sequence (5'-3')	Amplicon size (bp)	Concentration (nM)
*E*. *granulosus s*.*s*.	*nad*1	F-Eg	GGTTTTATCGGTATGTTGGTGTTAGTG	219	100
		R-Eg	CATTTCtTGAAGTTAACAGCATCACG		
*E*. *multilocularis*	*nad*5	F-Em	CATTAATTATGGATGTTTCC	584	50
		R-Em	GGAAATACCCCACTATCC		
*E*. *shiquicus*	*cox*1	F-Es	GCTTTAAGTGCGTGACTTTTAATCCC	471	100
		R-Es	CATCAAAACCAGCACTAATACTCA		

### Multiplex PCR assay

PCR amplification was carried out in a 25 μl mixture containing 2 μl dNTPs (2.5 mM of each), 2.5 μl 10× Ex*Taq* Buffer (Mg^2+^ free), 2 μl MgSO_4_ (25 mM), 0.25 μl Ex*Taq* DNA polymerase (5U/μl) (TaKaRa, Dalian, Liaoning), 100 pg DNA template of each *Echinococcus* sample, and all three primer pairs were added according to the final concentrations given in Table 2. Fragments were amplified using the following optimized thermocycling conditions: 95°C/5 min for denaturation; 30 cycles of 94°C/30 sec, 55°C/30 sec, 72°C/40 sec; and 72°C/10 min extension. For all the multiplex PCR assays, positive DNA (DNA templates of the three *Echinococcus* spp.) and negative (no-DNA) controls were included.

### Identification of PCR products

Amplicons were visualized by electrophoresis in 2.0% (w/v) agarose gels in 1×TAE (40 mM Tris-acetate, 2 mM EDTA, pH 8.5), stained with ethidium bromide (EB), and viewed under UV light. The fragments were purified using an agarose Gel DNA Purification Kit (TaKaRa, Dalian, Liaoning), and then cloned into pMD18-T Simple vectors using a TA cloning strategy. The recombinant vectors were identified by enzyme digestion and at least two clones for each DNA region were sequenced by the Shanghai Invitrogen Biotechnology Co. Ltd.

### Controls for the multiplex PCR assay

#### Positive control for fecal sample tests

DNA from protoscoleces of the three *Echinococcus* spp. was added to a fecal sample as positive control. Another type of positive control was provided by the copro-samples that were directly collected from dogs successfully infected with *E*. *multilocularis* or *E*. *granulosus s*.*s*.

#### Negative controls

To exclude the possibility of contamination in the PCR amplification, two negative fecal samples (no-DNA) were used. Other negative controls included all reagents except for the addition of parasite DNA.

#### Fecal inhibitor controls

To test for potential inhibitors, DNA extracted from protoscoleces and identified by gene sequencing was added to a negative fecal sample, and subjected to the multiplex PCR in parallel with the negative fecal sample.

#### Host tissue controls

DNAs extracted from tissues of dogs and foxes as well as those from intermediate hosts were tested using the multiplex PCR assay to determine the minimum contamination level that could cause interference in the assay. Host DNA (0.1, 0.5, 1, 5, 10, 50, 100, 500 or 1000 ng) was mixed into each relevant parasite DNA sample prior to the assay.

### Specificity and sensitivity

#### Specificity

Three pairs of primers were added to each PCR tube with the optimized multiplex PCR reaction conditions (described above) to test various parasite DNA samples as listed in Table 1.

#### Lowest/highest detection limit of DNA using *Echinococcus* larval tissue

DNA samples from protoscoleces of the three *Echinococcus* spp. were quantified by spectrophotometry using a NanoDrop 2000 (Thermo Scientiific, Wilmington, DE, USA). Serial dilutions of the DNA template (0.01, 0.02, 0.05, 0.1, 0.5, 1, 5, 10, 50, 100, 500 and 1000 ng) were used to assay the analytical sensitivity and potential nonspecific amplification of DNA in the multiplex PCR system. Amplification results were visualized by electrophoresis in a 2.0% (w/v) agarose gel.

#### Minimum numbers of eggs detectable in fecal samples

One to ten *Echinococcus* eggs were added to the diluted negative fecal samples. DNA extracted from these samples was used in the multiplex reaction to determine the minimum number of eggs that could yield a positive PCR outcome.

#### Earliest time post-infection on which dog fecal samples yielded positive PCR results

All copro-DNAs, extracted from fecal samples that had been collected every day from experimentally infected dogs, were tested using the multiplex PCR assay to determine the first day when a positive signal occurred.

## Results

### Infection outcomes of the experimentally challenged dogs and the examination of stray dogs

Infections of *E*. *granulosus s*.*s*. and *E*. *multilocularis* were successfully achieved in all the experimentally infected dogs with 5539, 8562, 12535, 18932 and 20775 *E*. *granulosus s*.*s*. and 2893, 3153, 3762, 3864 and 5322 *E*. *multilocularis* adult worms being recovered from each group of 5 dogs that were fed with protoscoleces of each species. No adult worms were found in any of the 5 dogs fed larval *E*. *shiquicus*. None of the stray dogs was found harboring *E*. *shiquicus* or *E*. *multilocularis*; only *E*. *granulosus s*.*s*. adult worms were found in their intestinal contents (identity confirmed by both morphology and *cox*1 sequencing). Worm burdens were relatively low (circa 100–200 worms) in the ten stray dogs examined.

### Identification of PCR products

**Fig 1:**
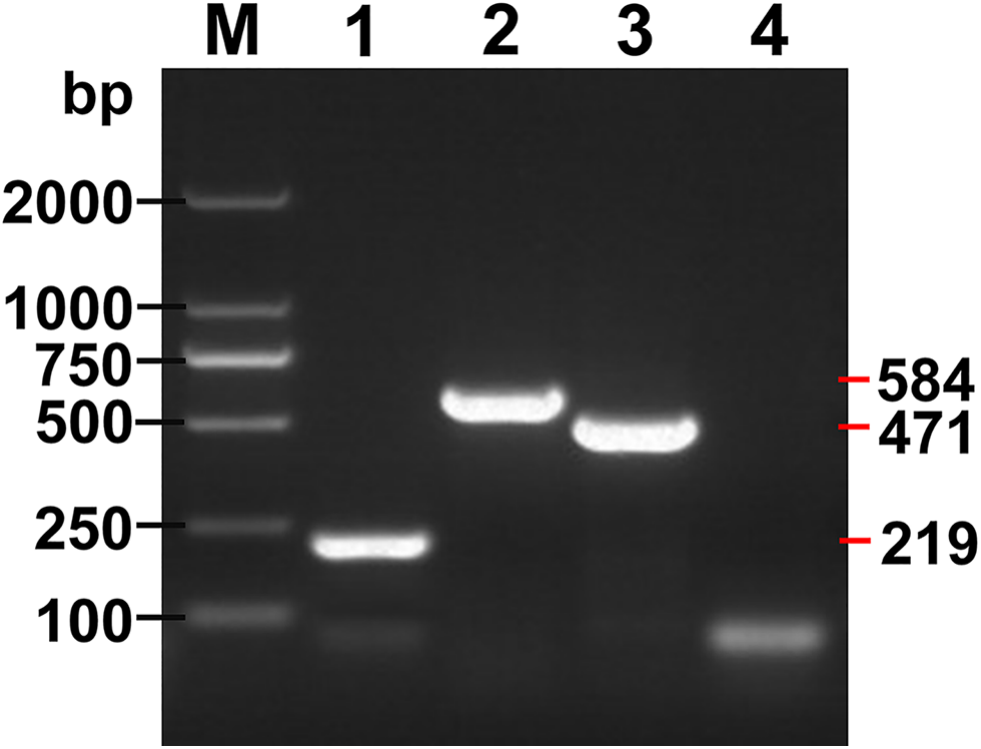
Amplicons of the target genes using the multiplex PCR assay. Lanes 1, 2 and 3, Amplicons of *E*. *granulosus s*.*s*., *E*. *multilocularis* and *E*. *shiquicus* respectively; Lane 4, Negative control; M, DNA Marker DL 2000.

**Fig 2:**
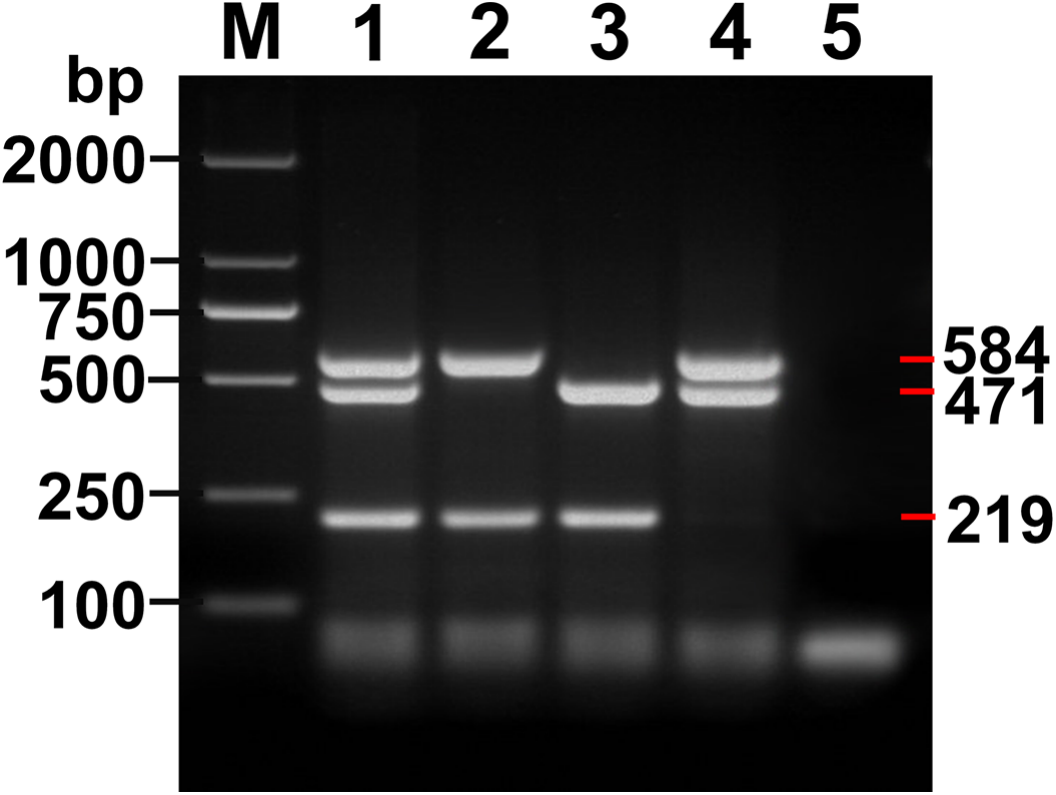
Amplicons of the mixed templates using the multiplex PCR assay. Lane 1, Amplicons of *E*. *granulosus s*.*s*., *E*. *multilocularis* and *E*. *shiquicus*; Lane 2, Amplicons of *E*. *granulosus s*.*s*. and *E*. *multilocularis*, Lane 3, Amplicons of *E*. *granulosus s*.*s*. and *E*. *shiquicus*; Lane 4, Amplicons of *E*. *granulosus s*.*s*., *E*. *multilocularis* and *E*. *shiquicus*; Lane 5, Negative control; M, DNA Marker DL 2000.

Expected PCR products of 219, 584 and 471 bp were obtained for *E*. *granulosus s*.*s*. (*nad*1), *E*. *multilocularis* (*nad*5) and *E*. *shiquicus* (*cox*1), respectively (Fig 1), and products of mixed templates of the three *Echinococcus* species are shown in Fig 2. The multiplex PCR products contained 3 DNA bands (219, 471 and 584 bp) with mixed DNA templates of *E*. *granulosus s*.*s*., *E*. *multilocularis* and *E*. *shiquicus*; 2 DNA bands (219 and 584 bp) with *E*. *granulosus s*.*s*. and *E*. *multilocularis* DNA templates; 2 DNA bands (219 and 471 bp) with *E*. *granulosus s*.*s*. and *E*. *shiquicus* DNA templates; and 2 DNA bands (471 and 584 bp) with *E*. *multilocularis* and *E*. *shiquicus* DNA templates. DNA sequences of these products corresponded in each case with the relevant reference sequences in GenBank: *E*. *granulosus* (G1) (NC_008075) [22], *E*. *multilocularis* (NC_000928) [23] and *E*. *shiquicus* (NC_009460) [24].

### Specificity

#### Comparison of various sources of DNA

**Fig 3:**
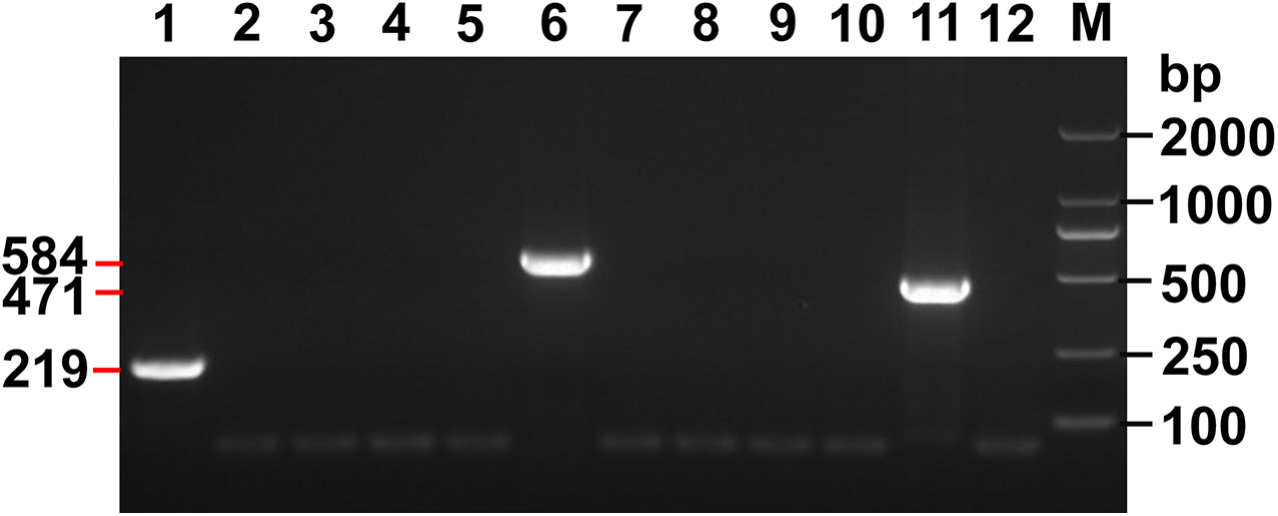
Specificity of the multiplex PCR assay. M, DNA Marker DL 2000; Lanes 1, 6 and 11, Amplicons of *E*. *granulosus s*.*s*., *E*. *multilocularis* and *E*. *shiquicus* respectively; Lane 2–5, 7–10 and 12, Other cestode samples: e.g. *E*. *canadensis* (G6), *T*. *hydatigena*, *T*. *multiceps*, *T*. *pisiformis*, *T*. *taeniaeformis*, *T*. *solium*, *D*. *caninum*, liver tissue of Qinghai vole, lung tissue of plateau pika.

No PCR products were detected when DNAs from other cestodes (Table 1, labeled with an asterisk) were used in the multiplex assay (Fig 3). False positive results were never produced from confirmed negative samples. Further, no PCR products were obtained when DNA samples from various host tissues were used in the multiplex PCR. Therefore, the specificity of the multiplex PCR for *E*. *granulosus s*.*s*. (G1), *E*. *multilocularis* and *E*. *shiquicus* was shown to be 100%.

#### Copro-DNA templates

Fecal samples, collected from dogs before experimental infection with *Echinococcus* spp. and from captive foxes (confirmed parasite-free by microscopy and DNA analysis), were negative in the multiplex PCR whereas fecal samples from dogs after experimental infection with either larval *E*. *granulosus s*.*s*. or *E*. *multilocularis* were positive. Furthermore, the PCR products obtained were of the expected sizes, matching those that were also obtained for all positive controls. No false positive signals were obtained with any negative control sample.

#### Fecal inhibitors and the copro-DNA test

To test for the presence of potential inhibitors, parallel multiplex PCR assays were performed with positive *Echinococcus* spp. DNA, negative fecal samples, and mixtures of *Echinococcus* spp. DNA and fecal sample DNA as templates. Positive signals were detectable in the multiplex PCRs with mixtures of the *Echinococcus* spp. DNAs and fecal sample DNA as templates. We infer from this that no adverse fecal inhibitors affected the integrity of the multiplex PCR assay.

#### Effect of host tissue DNA on the multiplex PCR test

To test for interference due to host DNA contamination in samples, we added various quantities of host DNA to known quantities (100 pg) of parasite DNA. Quantities below 500 ng of host tissue DNA (from intestinal, hepatocyte or pulmonary cells) did not affect PCR outcomes: clear bands of expected sizes were present in gels. However, smeared bands appeared in the gels if the amount of host DNA exceeded 500 ng.

### Sensitivity

#### Minimum/maximum quantity of *Echinococcus* metacestode DNA

The lower limit for the detection of metacestode DNA was 20 pg for *E*. *granulosus s*.*s*., 10 pg for *E*. *multilocularis*, and 20 pg for *E*. *shiquicus*, respectively. Clear bands could be visualized up to a maximum quantity of 500 ng template DNA. Smearing of bands occurred if this amount was exceeded. Accordingly, the optimum amount of template DNA used was 100 pg for the multiplex PCR as this quantity produced clear amplicon bands and provided savings on template DNA and PCR primers.

#### Minimum number of eggs detectable in the fecal samples

**Fig 4:**
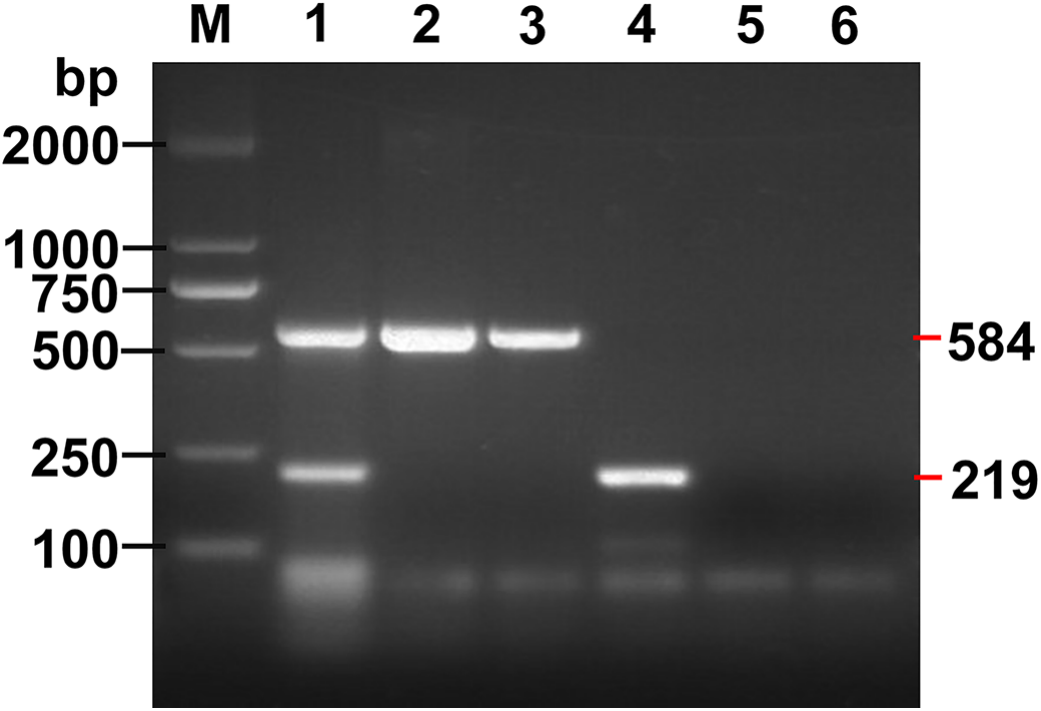
Sensitivity of the multiplex PCR assay. Lane 1, Amplicons of *E*. *granulosus s*.*s*. and *E*. *multilocularis* with 2 eggs and 1 egg respectively, mixed in fecal sample; Lanes 2 and 3, Amplicons of *E*. *multilocularis* from 2 eggs and 1 egg respectively, mixed in fecal sample; Lanes 4 and 5, Amplicon of *E*. *granulosus s*.*s*. amplified with 2 eggs and 1 egg mixed in fecal sample; Lane 6, Negative control fecal sample; M, DNA Marker DL 2000.

Positive PCR products were obtained in reactions using DNA from as few as two eggs of *E*. *granulosus s*.*s*. and one egg of *E*. *multilocularis* (Fig 4).

#### Earliest time for a positive multiplex PCR assay after experimental infection

Eggs of *E*. *granulosus s*.*s*. and *E*. *multilocularis* were visualized under microscopy at days 47–56 and days 36–44 post-challenge, respectively. The multiplex PCR assay yielded positive results from copro-DNA 17 days after experimental infection of dogs with larval *E*. *multilocularis* and 26 days after infection with larval *E*. *granulosus s*.*s*.

#### Assessment of feces from stray dogs

Copro-DNA from all the stray dogs infected with *E*. *granulosus s*.*s*. tested positive in the multiplex PCR.

## Discussion

China is the most severe pandemic country for cystic echinococcosis (CE), in humans and livestock, due mainly to *E*. *granulosus s*.*s*., and for alveolar echinococcosis (AE) due to *E*. *multilocularis* in humans and small wild mammals. *E*. *shiquicus* is also endemic although it has not been reported to infect humans. Dual infections of animal hosts with different *Echinococcus* spp have been reported in the eastern Qinghai-Tibet plateau region of China [4, 25]. The very close relationship between dogs and humans can lead readily to human infection. The increasing number of human AE and CE cases in northwestern China, where considerable numbers of dogs are present, places a heavy burden on public health and veterinary services. To aid surveillance, management and diagnosis, effective methods are needed for rapid and accurate detection and identification of different life cycle stages of the three *Echinococcus* spp. simultaneously. The multiplex PCR assay developed in this study provides such a method.

Traditional epidemiological surveys for tapeworms often involve recovery of eggs from feces of potential definitive hosts. However, morphological identification of *Echinoccocus* eggs to species level is practically impossible, prompting the development of several molecular approaches [26, 27]. Inhibitors present in fecal material that co-purify with parasite DNA extracted from feces often present a problem for PCR-based methods [28]. In this study, the QIAGEN QIAamp DNA Stool Mini Kit, containing InhibitEX tablets for removing inhibitors in fecal samples, was used to purify copro-DNA. The sieving-flotation method was helpful in overcoming this problem due to its enrichment of worm eggs [29]. The positive control (protoscolex DNA in fecal samples) used in this study demonstrated the lack of inhibitor effects in our copro-multiplex PCR assay.

*E*. *granulosus s*.*s*. has been reported as having a pre-patent period of 6 weeks (42 days) [30, 31], while *E*. *multilocularis* eggs have been observed in feces at 42–46 days post infection [32]. However, in the current study we first identified eggs of *E*. *granulosus s*.*s*. at 47–56 days post-challenge and those of *E*. *multilocularis* at 36–44 days post-challenge by microscopy similar to reports by others [30, 33]. The discrepancies between these studies may be due to the use of different dog-breeds, ages, nutrient status or the conditions under which the dogs were maintained. We were unable to experimentally infect dogs with *E*. *shiquicus* although the viability of the challenge sample of protoscoleces was confirmed by microscopy.

PCR-positive signals in this study were obtained from dog fecal samples much earlier (17 days for *E*. *multilocularis* and 26 days for *E*. *granulosus*) than any other previous studies using microscopy as a method of detecting infected canid hosts. The much earlier detection of an *Echinococcus* infection by the multiplex PCR method compared with egg recovery from feces and microscopic examination is a marked improvement that can aid surveillance programs aimed at preventing echinococcosis transmission.

The method developed in this study has achieved high species specificity because it produced no amplicon from any other helminth (including several that might dual infect with *Echinococcus* species in dogs) or from the negative copro-samples (no-DNA). The primer set (three pairs of primers) multiplex reaction in a single tube worked well with all templates tested and yielded specific amplicons of the expected length for each of the three *Echinococcus* spp. examined.

*E*. *granulosus s*.*s* and *E*. *multilocularis* are of major public health concern in many endemic countries globally [34]. A cost effective diagnostic tool is required for echinococcosis surveillance of definitive and intermediate hosts, and for monitoring the effectiveness of control programs. The multiplex PCR assay developed in this study provides an effective method that can be applied in both clinical and epidemiological settings for the identification of *Echinococcus* spp in diverse hosts, and would be particularly useful for identifying infected hosts in areas co-endemic for AE and CE.

In this study, we focused on *Echinococcus* samples collected from the Qinghai-Tibet plateau region of China, where three species (*E*. *granulosus*, *E*. *multilocularis* and *E*. *shiquicus*) are known to be endemic. In total, nine species are now recognized in the genus *Echinococcus*, including *E*. *granulosus sensu stricto* (genotypes G1-G3), *E*. *equinus*, *E*. *canadensis* (genotypes G6, G7, G8 and G10), *E*. *ortleppi*, *E*. *multilocularis*, *E*. *shiquicus*, *E*. *vogeli*, *E*. *oligarthrus* and *E*. *felidis* [1]. None of the three specific pairs of primers developed in this study produced a PCR-amplified product using DNA isolated from *E*. *canadensis* (G6 genotype) showing in Fig 3 (the lane 2 with non-band as a negative result). This is supported by inspection and comparison of the primer target sequence for the G6 genotype with those of the three *Echinococcus* spp., which showed six base pair differences between them (S1 Fig, S2 Fig and S3 Fig in the Supporting Supplementary Information). Furthermore, six or more base pair differences are apparent between the target sequences for *E*. *equinus*, *E*. *canadensis* (genotypes G7, G8 and G10), *E*. *ortleppi*, *E*. *vogeli*, *E*. *oligarthrus* and *E*. *felidis*. Therefore, it is highly unlikely that any amplicon would be produced from these species during the multiplex PCR due to its high species specificity.

## Supporting Information

S1 Checklist STARD Checklist. (DOC)

S2 Checklist Flowchart. (DOCX)

S1 Fig Primer design for *Echinococcus granulosus sensu stricto*. (PDF)

S2 Fig Primer design for *Echinococcus multilocularis*. (PDF)

S3 Fig Primer design for *Echinococcus shiquicus*. (PDF)
