# Supplementary material for: Discrimination between E. granulosus sensu stricto, E. multilocularis and E. shiquicus Using a Multiplex PCR Assay
Source: PLoS Negl Trop Dis. 2015 Sep 22;9(9):e0004084. doi: 10.1371/journal.pntd.0004084 (PMC4578771; doi:10.1371/journal.pntd.0004084)
Supplement: S1 Checklist — (DOC) [file pntd.0004084.s001.doc]

# STARD checklist for discrimination between *E. granulosus* *sensu stricto*, *E. multilocularis* and *E. shiquicus* using a multiplex PCR assay

| **Section and Topic** | **Item**  **#** |  | **On page #** |
| --- | --- | --- | --- |
| TITLE/ABSTRACT/  KEYWORDS | 1 | Discrimination between E. granulosus sensu stricto, E. multilocularis and E. shiquicus using a multiplex PCR assay/  The multiplex PCR enabled diagnosis of multiple infections using DNA of protoscoleces and copro-DNA extracted from fecal samples of canine hosts. Specificity of the multiplex PCR was 100% when evaluated using DNA isolated from other cestodes. Sensitivity thresholds were determined for DNA from protoscoleces and from worm eggs, and were calculated as 20 pg of DNA for *E. granulosus* and *E. shiquicus*, 10 pg of DNA for *E. multilocularis*, 2 eggs for *E. granulosus*, and 1 egg for *E. multilocularis*. Positive results with copro-DNA could be obtained at day 17 and day 26 after experimental infection of dogs with larval *E. multilocularis* and *E. granulosus*, respectively./  Multiplex PCR assay, DNA diagnosis | 1-3 |
| INTRODUCTION | 2 | Multiplex PCR approaches, simultaneously using multiple specific primers in a single tube and detecting more than one target species, are material- and time-saving, precise, efficient and cost-effective when DNA from a mixture of pathogens may be present in a sample. This approach is also suitable for mass-screening of samples that may be generated from epidemiological investigations in endemic areas. Based on interspecific variation in mitochondrial genes of the genus Echinococcus, we designed a multiplex PCR assay with three pairs of specific primers in a single reaction tube for rapid identification of E. granulosus s.s., E. multilocularis and E. shiquicus originating from either intermediate or definitive hosts. Further assessment of the sensitivity and specificity of the multiplex PCR assay was performed using metacestode DNA and copro-DNA to determine the reliability and accuracy of the new diagnostic tool developed in this study. | 4 |
| METHODS |  |  |  |
| *Participants* | 3 | **Sampling of *Echinococcus* material**  Adult worms were collected from stray dogs during routine work of the endemic echinococcosis prevention and control program in Dari County, Qinghai Province, P.R. China. A total of 86 Echinococcus spp. metacestode samples from yaks, sheep, Qinghai voles (Microtus/Neodon fuscus) and plateau pikas were collected on the Qinghai-Tibet plateau, P.R. China. Ten yak lungs and 16 sheep livers harboring hydatid cysts were collected from abattoirs in Maqu County, Gansu Province and Xining City, Qinghai Province, respectively. Thirty Qinghai vole livers and 30 plateau pika lungs harboring hydatid cysts were provided by the epidemic prevention station of Dari County, Qinghai Province.  **Sampling of adults/eggs of *Echinococcus* spp. from challenged dogs**  Fecal samples were collected from the dogs each day prior to sacrifice. After removal of the coarse gut contents, the small intestine was cut into 15-20 cm lengths and opened to expose the mucosa. Samples, taken by scraping the mucosa with glass strips, were placed in petri dishes in bio-safety containers. Adult worms were removed using a glass needle and washed in PBS three times.  **Fecal sampling from non-experimented definitive hosts**  Ten stray dogs, provided by the epidemic prevention station in Dari County, Qinghai Province, were processed as above to obtain mucosal samples, worms and eggs. Additionally, five fecal samples from captive foxes were collected from a fur farm in Lanzhou City, Gansu Province. **Other helminths**  DNA samples, extracted from a variety of cestodes (identities confirmed by sequencing and morphology), were used to determine the specificity of the newly developed multiplex PCR assay. They were kindly provided by the Key Laboratory of Veterinary Parasitology of Gansu Province, Lanzhou Veterinary Research Institute, CAAS.  **Host tissue sampling**  Host tissues included dog intestines, and liver and lung samples from cattle, sheep, Qinghai voles and plateau pikas. | 5-7 |
|  | 4 | **Participant recruitment:** Recruitment was based on presenting symptoms, results from previous tests, and the fact that the participants had received the index tests. |  |
|  | 5 | **Participant sampling:** The study population was a consecutive series of participants defined by the selection criteria in item 3 and 4. |  |
|  | 6 | **Data collection:** Data collection was planned before the index test. |  |
| *Test methods* | 7 | Multiplex PCR approaches, simultaneously using multiple specific primers in a single tube and detecting more than one target species, are material- and time-saving, precise, efficient and cost-effective when DNA from a mixture of pathogens may be present in a sample. This approach is also suitable for mass-screening of samples that may be generated from epidemiological investigations in endemic areas. | 4-5 |
|  | 8 | **Multiplex PCR assay**  PCR amplification was carried out in a 25 μl mixture containing 2 μl dNTPs (2.5 mM of each), 2.5 μl 10× Ex*Taq* Buffer (Mg^2+^ free), 2 μl MgSO_4_ (25 mM), 0.25 μl Ex*Taq* DNA polymerase (5U/μl) (TaKaRa, Dalian, Liaoning), 100 pg DNA template of each *Echinococcus* sample, and all three primer pairs were added according to the final concentrations given in Table 2. Fragments were amplified using the following optimized thermocycling conditions: 95℃/ 5 min for denaturation hold; 30 cycles of 94℃/ 30 sec, 55℃/ 30 sec, 72℃/ 40 sec; and 72℃/ 10 min extension holding. For all the multiplex PCR assays, positive DNA (DNA templates of the three species of *Echinococcus*) and negative (no-DNA) controls were included.  **Specificity and sensitivity**  **Specificity.** Three pairs of primers were added to each PCR tube with the optimized multiplex PCR reaction conditions (described above) to test various parasite DNA samples as listed in Table 1.  **Lowest/highest detection limit of DNA using *Echinococcus* larval tissue.** DNA samples from protoscoleces of the three species of *Echinococcus* were quantified by spectrophotometry using a NanoDrop 2000 (Thermo Scientiific, Wilmington, DE, USA). Serial dilutions of the DNA template (0.01, 0.02, 0.05, 0.1, 0.5, 1, 5, 10, 50, 100, 500 and 1000 ng) were used to assay the analytical sensitivity and potential nonspecific amplification of the multiplex PCR system. Amplification results were visualized by electrophoresis in a 2.0% (w/v) agarose gel.  **Minimum numbers of eggs detectable in fecal samples.** One to ten *Echinococcus* eggs were added to the diluted negative fecal samples. DNA extracted from these samples was used in the multiplex reaction to determine the minimum number of eggs that could yield a positive PCR outcome.  **Earliest day post-infection on which dog fecal samples yielded positive PCR results.** All copro-DNAs, extracted from fecal samples that had been collected every day from experimentally infected dogs, were tested using the multiplex PCR assay to determine the first day when a positive signal occurred. | 8-10 |
|  | 9 | Amplicons were visualized by electrophoresis in 2.0% (w/v) agarose gels in 1×TAE (40 mM Tris-acetate, 2 mM EDTA, pH 8.5), stained with ethidium bromide (EB), and viewed under UV light. | 9 |
|  | 10 |  |  |
|  | 11 |  |  |
| *Statistical methods* | 12 |  |  |
|  | 13 |  |  |
| RESULTS |  |  |  |
| *Participants* | 14 | Infections of E. granulosus s.s. and E. multilocularis were successfully achieved in all the experimentally infected dogs with 5539, 8562, 12535, 18932 and 20775 E. granulosus s.s. and 2893, 3153, 3762, 3864 and 5322 E. multilocularis adult worms being recovered from each group of 5 dogs that were fed with protoscoleces of each species. No adult worms were found in any of the 5 dogs fed larval E. shiquicus. None of the stray dogs was found harboring E. shiquicus or E. multilocularis; only E. granulosus s.s. adult worms were found in their intestinal contents (identity confirmed by both morphology and cox1 sequencing). Worm burdens were relatively low (circa 100-200 worms) in the ten stray dogs examined. | 9-10 |
|  | 15 |  |  |
|  | 16 |  |  |
| *Test results* | 17 |  |  |
|  | 18 |  |  |
|  | 19 | **Identification of PCR products**  Expected PCR products of 219, 584 and 471 bp were obtained for E. granulosus s.s. (nad1), E. multilocularis (nad5) and E. shiquicus (cox1), respectively. The multiplex PCR products contained 3 DNA bands (219, 471 and 584 bp) with mixed DNA templates of E. granulosus s.s., E. multilocularis and E. shiquicus; 2 DNA bands (219 and 584 bp) with E. granulosus s.s. and E. multilocularis DNA templates; 2 DNA bands (219 and 471 bp) with E. granulosus s.s. and E. shiquicus DNA templates; and 2 DNA bands (471 and 584 bp) with E. multilocularis and E. shiquicus DNA templates.  **Specificity**  **Comparison of various sources of DNA.**  False positive results were never produced from confirmed negative samples. Further, no PCR products were obtained when DNA samples from various host tissues were used in the multiplex PCR. Therefore, the specificity of the multiplex PCR for E. granulosus s.s. (G1), E. multilocularis and E. shiquicus was shown to be 100%.  **Copro-DNA templates.** Fecal samples, collected from dogs before experimental infection with Echinococcus spp. and from captive foxes (confirmed parasite-free by microscopy and DNA analysis), were negative in the multiplex PCR whereas fecal samples from dogs after experimental infection with either larval E. granulosus s.s. or E. multilocularis were positive. Furthermore, the PCR products obtained were of the expected sizes, matching those that were also obtained for all positive controls. No false positive signals were obtained with any negative control sample.  **Effect of host tissue DNA on the multiplex PCR test.** Quantities below 500 ng of host tissue DNA (from intestinal, hepatocyte or pulmonary cells) did not affect PCR outcomes: clear bands of expected sizes were present in gels. However, smeared bands appeared in the gels if the amount of host DNA exceeded 500 ng.  **Sensitivity**  **Minimum/maximum quantity of Echinococcus metacestode DNA.** The lower limit for the detection of metacestode DNA was 20 pg for E. granulosus s.s., 10 pg for E. multilocularis, and 20 pg for E. shiquicus, respectively. Clear bands could be visualized up to a maximum quantity of 500 ng template DNA. Smearing of bands occurred if this amount was exceeded.  **Minimum number of eggs detectable in the fecal samples.** Positive PCR products were obtained in reactions using DNA from as few as two eggs of E. granulosus s.s. and one egg of E. multilocularis.  **Earliest time for a positive multiplex PCR assay after experimental infection.** Eggs of E. granulosus s.s. and E. multilocularis were visualized under microscopy at days 47-56 and days 36-44 post-challenge, respectively. The multiplex PCR assay yielded positive results from copro-DNA 17 days after experimental infection of dogs with larval E. multilocularis and 26 days after infection with larval E. granulosus s.s. | 10-13 |
|  | 20 | No |  |
| *Estimates* | 21 |  |  |
|  | 22 |  |  |
|  | 23 |  |  |
|  | 24 |  |  |
| DISCUSSION | 25 | To aid surveillance, management and diagnosis, effective methods are needed for rapid and accurate detection and identification of different life cycle stages of the three Echinococcus spp. simultaneously. The multiplex PCR assay developed in this study provides such a method.  In the current study we first identified eggs of E. granulosus s.s. at 47-56 days post-challenge and those of E. multilocularis at 36-44 days post-challenge by microscopy similar to reports by others [30, 33]. The discrepancies between these studies may be due to the use of different dog-breeds, ages, nutrient status or the conditions under which the dogs were maintained. We were unable to experimentally infect dogs with E. shiquicus although the viability of the challenge sample of protoscoleces was confirmed by microscopy.  PCR-positive signals in this study were obtained from dog fecal samples much earlier (17 days for E. multilocularis and 26 days for E. granulosus) than any other previous studies using microscopy as a method of detecting infected canid hosts. The much earlier detection of an Echinococcus infection by the multiplex PCR method compared with egg recovery from feces and microscopic examination is a marked improvement that can aid surveillance programs aimed at preventing echinococcosis transmission.  The method developed in this study has achieved high species specificity because it produced no amplicon from any other helminth (including several that might dual infect with Echinococcus species in dogs) or from the negative copro-samples (no-DNA). The primer set (three pairs of primers) multiplex reaction in a single tube worked well with all templates tested and yielded specific amplicons of the expected length for each of the three Echinococcus spp. examined.  The multiplex PCR assay developed in this study provides an effective method that can be applied in both clinical and epidemiological settings for the identification of Echinococcus spp in diverse hosts, and would be particularly useful for identifying infected hosts in areas co-endemic for AE and CE.  None of the three specific pairs of primers developed in this study produced a PCR-amplified product using DNA isolated from E. canadensis (G6 genotype). This is supported by inspection and comparison of the primer target sequence for the G6 genotype with those of the three Echinococcus spp., which showed six base pair differences between them.  Furthermore, as shown in the Supporting Supplementary Information (Figures S1, S2 and S3), six or more base pair differences are apparent between the target sequences for E. equinus, E. canadensis (genotypes G7, G8, G10), E. ortleppi, E. vogeli, E. oligarthrus and E. felidis. Therefore, it is highly unlikely that any amplicon would be produced from these species during the multiplex PCR due to its high species specificity. | 14-15 |
