# Supplementary material for: Discrimination between E. granulosus sensu stricto, E. multilocularis and E. shiquicus Using a Multiplex PCR Assay
Source: PLoS Negl Trop Dis. 2015 Sep 22;9(9):e0004084. doi: 10.1371/journal.pntd.0004084 (PMC4578771; doi:10.1371/journal.pntd.0004084)
Supplement: S2 Checklist — (DOCX) [file pntd.0004084.s002.docx]

Flowchart for discrimination between *E. granulosus* *sensu stricto*, *E. multilocularis* and *E. shiquicus* in China using a multiplex PCR assay

Samples of *E. granulosus sensu stricto, E. multilocularis and E. shiquicus* for analysis

471 and 584 bp bands with *E. multilocularis* and *E. shiquicus* respectively

219 and 471 bp bands with *E. granulosus s.s*. and *E. shiquicus* respectively

219 and 584 bp bands with *E. granulosus s.s*. and *E. multilocularis* respectively

219, 471 and 584 bp bands with mixed DNA templates of *E. granulosus s.s*., *E. multilocularis* and *E. shiquicus* respectively

Positive results; Sensitivity thresholds were 20 pg of DNA for *E. granulosus* and *E. shiquicus*, 10 pg of DNA for *E. multilocularis*, 2 eggs for *E. granulosus*, and 1 egg for *E. multilocularis*; Specificity was 100%.

Fecal samples (test and field) from canids

Multiplex PCR assays performed with different DNA templates

Adult worms (test and field)

Stool flotation in saturated zinc chloride solution

Metacestode (field samples)

Parasite materials

Negative

No band

219, 471 or 584 bp band for DNA templates of *E. granulosus s.s*., *E. multilocularis* or *E. shiquicus* respectively

Genomic DNA extraction using a QIAGEN QIAamp DNA Stool Mini Kit

Genomic DNA extraction using a QIAGEN DNeasy Blood & Tissue Kit
