# Supplementary material for: Discrimination between E. granulosus sensu stricto, E. multilocularis and E. shiquicus Using a Multiplex PCR Assay
Source: PLoS Negl Trop Dis. 2015 Sep 22;9(9):e0004084. doi: 10.1371/journal.pntd.0004084 (PMC4578771; doi:10.1371/journal.pntd.0004084)
Supplement: S1 Fig — (PDF) [file pntd.0004084.s003.pdf]

S1 Fig: Primer design for *Echinococcus granulosus sensu stricto*

F-Eg

```
                  5′-GGTTTTATCGGTATGTTGGTGTTAGTG-3′
E.granulosus         GGTTTTATCGGTATGTTGGTGTTAGTGTTTAATTTATTATTTTTTATGTGGGCTCGGGCG
E.canadensis G7      GGTATTGTTGGTATGTTCATGTTGGTGTTTAATTTACTGTTTTTTATGTGGGCTCGAGCG
E.canadensis G6      GGTATTGTTGGTATGTTCATGTTGGTGTTTAATTTACTGTTTTTTATGTGGGCTCGAGCG
E.canadensis G10     GGTATTGTTGGTATGTTCATGTTGGTGTTTAATTTACTGTTTTTTATGTGGGCTCGAGCT
E.canadensis G8      GGTATTGTTGGTATGTTTATGTTGGTGTTTAATTTACTGTTTTTTATGTGGGCTCGAGCG
E.ortleppi           GGTATTGTTGGTATGTTTATGTTGATGTTTAATTTATTGTTTTTTATGTGGGCTCGAGCG
E.multilocularis     GGTTTTGTTGGTTTGTTTATGTTGTTGTTTAATTTATTATTTTTTATGTGAGCTCGGGCA
E.shiquicus          GGTATGGTTGGGTTGTTTTTGTTGGTATTCAATTTATTGTTTTTTATGTGAGCTCGTGCG
E.equinus            GGCTTTGTTGGTATGTTTATGTTGTTATTTAATTTATTGTTTTTTATGTGGACTCGTGCA
E.vogeli             GGAGGTGTTGGTATGTTGATGCTGATGTTTAATCTATTATTTTTTATGTGGGCTCGGGCG
E.oligarthrus        GGTAAGGTTGCTATGATTATGTTGATGTTTAATTTATTATTTTTTATGTGAGCTCGGGCT
E.felidis            GGTTTTATTGGTATGTTGATGCTTATGTTTAATTTATTATTTTTGATGTGAGCTCGGGCT
                     **    .* *  :**:*  ** *  *.** *** ** *.***** *****..**** **
```

R'-Eg

```
                                        5′-CGTGATGCTGTTAACTTCAAGAAATG-3′
E.granulosus         GTCTGTATAGATTATC-GAAATCGTGATGCTGTTAACTTCAAGAAATGGTTATCGCCATT
E.canadensis G7      GTCTGTATAGATTATT-AAAATCGTGATGCTGTTAACTTCAAGAAATGGTTATTACCATT
E.canadensis G6      GTCTGTATAGATTATT-AAAATCGTGATGCTGTTAACTTCAAGAAATGGTTATTACCATT
E.canadensis G10     GTCTGTATAGATTATT-AAAATCGTGATGCTGTTAACTTCAAGAAATGGTTATTACCATT
E.canadensis G8      GTCTGTATAGATTATT-AAAATCGTGATGCTGTTAACTTCAAGAAATGGTTATTACCATT
E.ortleppi           GTCTGTATAGATTATT-AAAATCGTGATGCTGTTAACTTCAAGAAATGGTTATTGCCATT
E.multilocularis     --CTGTATAGATTATT-TAAATCGTGATGCTGTTAACTTCAAGAAATGGTTATTTCCATT
E.shiquicus          GTCTGTATAGATTATTCTAAATCGTGATGCTGTTAACTTCAAGAAATGGTTATTGCCATT
E.equinus            GTCTATATAGATTATT-AAAATCGTGATGCTGTTAACTTCAAGAAATGGTTATAGCCATT
E.vogeli             GTCCGTATAGATTATT-GAAATCGTGATGCTGTTAACTTCAAGAAATGGTTATTGCCATT
E.oligarthrus        GTCCGTATAGATTATT-AAAATCGTGATGCTGTTAACTTCAAGAAATGGTTATTGCCATT
E.felidis            GTCTGTATAGATTATT-CAAATCGTGATGCTGTTAACTTCAAGAAATGGTTATTGCCATT
                       * .**********   ***********************************  *****
```
