# Supplementary material for: Discrimination between E. granulosus sensu stricto, E. multilocularis and E. shiquicus Using a Multiplex PCR Assay
Source: PLoS Negl Trop Dis. 2015 Sep 22;9(9):e0004084. doi: 10.1371/journal.pntd.0004084 (PMC4578771; doi:10.1371/journal.pntd.0004084)
Supplement: S2 Fig — (PDF) [file pntd.0004084.s004.pdf]

S2 Fig: Primer design for *Echinococcus multilocularis*

F-Em

```
                           5′-CATTAATTATGGATG-TTTCC-3′
E.multilocularis        TTTAAGCATTAATTATGGATG-TTTCCTAGCTATT--------ATGGTGTTAGTTG----
E.granulosus            TTTAAGCATTAATTATGGATG-TTTCCTAGCTATT-------TTTATTGCG---------
E.felidis               TTTAAGCATTAATTATGGATG-TTTCCTGGCTATTTACTG--TTTGTTGAG---------
E.equinus               TTTAAGCATTAATTATGGAGG-TGTCCTGGCTATTTG-----TTTGTTGTGT--------
E.canadensis G7         TTTAAGCATTAATTATGGATG-TTTCCTAGCTATT--------TTGTCAT----------
E.canadensis G6         TTTAAGCATTAATTATGGATG-TTTCCTAGCTATT--------TTGTCAT----------
E.canadensis G10        TTTAAGCATTAATTATGGATG-TTTCCTAGCTATT--------TTGTAAT----------
E.canadensis G8         TTTAAGCATTAATTATGGATG-TTTCCTAGCTATT--------TTGTTAT----------
E.ortleppi              TTTAAGCATTAATTATGGATTGTTTCCTAGCTATT--------TTGTTAT----------
E.shiquicus             TTTAAGCATTAATTATGGATGTTTTCCTGGCTATT--------CTGTTGTTATCTTGTTG
E.vogeli                TTTAAGCATTAATTATGGAGG-TTTCCTGGCTATT--------TTGTGGTTTGTTG----
E.oligarthrus           TTTAAGCATTAATTATGGATG-TTTCCTGGCTATTTTGTTGATTTGTTGTTG--------
                        *******************   * ****.******         *.   .
```

R'-Em

```
                      5′-GGATAGTGGGGTATTTCC-3′
E.multilocularis        TGGATAGTGGGGTATTTCCTTGACTTGTATGTTTTTTTATGATTGTTTTTTCAAAGAGTG
E.granulosus            TTGACAGTGGGTGGTTTCCTTGACTTGTGTGTTTTTTTTTGGTAGTTTTTTCTAAGAGTG
E.felidis               TTGATAGTGGGTGGTTTCCTTGATTTGTGTGTTTTTTTTTGTTAATTTTTTCTAAGAGTG
E.equinus               TTGATAGTGGTGTTTTTCCTTGACTTGTGTGTTTTTTTCTGGTAGTCTTTTCTAAGAGTG
E.canadensis G7         TTTATGGTGGTGTTATTCCTTGATTTGTGTGCTTTTTTTTTATAATATTTTCTAAGAGTG
E.canadensis G6         TTTATGGTGGTGTTATTCCTTGATTTGTGTGCTTTTTTTTTATAATATTTTCTAAGAGTG
E.canadensis G10        TTTATGGTGGTGTTATTCCTTGATTTGTGTGCTTTTTTTTTATAATATTTTCTAAGAGTG
E.canadensis G8         TTTATGGTGGTGTTATTCCTTGACTTGTGTGCTTTTTTTTGATAATATTTTCTAAGAGTG
E.ortleppi              TTTACGGTGGTGTTATTCCTTGACTTGTGTGCTTTTTTTTGATAATATTTTCTAAGAGTG
E.shiquicus             TTGATAGTATGACTTTGCTATGACTTGTGTGTTTTTATTTGGTGGTTTTTTCTAAGAGTG
E.vogeli                TGTATGGTGGTAATTTTTTATGGCTTGTGTGTTTTTTTTTTAGTGGTTTTTTCTAAGAGTG
E.oligarthrus           TGGGTGTAGGTGGTTTATTTTTATTAATGTGTTTTTTTTTAGTCGTATTTTCTAAGAGTG
                        *  . .  :.      :*   :* .  *:.*.** ****:* *  * .* *****:*******
```
