# Supplementary material for: Discrimination between E. granulosus sensu stricto, E. multilocularis and E. shiquicus Using a Multiplex PCR Assay
Source: PLoS Negl Trop Dis. 2015 Sep 22;9(9):e0004084. doi: 10.1371/journal.pntd.0004084 (PMC4578771; doi:10.1371/journal.pntd.0004084)
Supplement: S3 Fig — (PDF) [file pntd.0004084.s005.pdf]

S3 Fig: Primer design for *Echinococcus shiquicus*

F-Es

```
                                5′-GCTTTAAGTGCGTGACTTTTAATCCC-3′
E.shiquicus          GAATTTGCCGCGTTTGAATGCTTTAAGTGCGTGACTTTTAATCCCTTCGTTGGTTTTGTT
E.canadensis G7      GAATTTACCGCGTTTGAATGCTTTGAGTGCGTGGCTTTTAGTTCCTTCGTTGGTTTTTTT
E.canadensis G6      GAATTTACCGCGTTTGAATGCTTTGAGTGCGTGGCTTTTAGTTCCTTCGTTGGTTTTTTT
E.canadensis G10     GAATTTACCGCGTTTGAATGCTTTGAGTGCGTGGCTTTTAGTTCCTTCATTGGTTTTTTT
E.canadensis G8      GAATTTACCGCGTTTGAATGCTTTGAGTGCGTGGCTTTTAGTTCCTTCGTTGGTTTTTTT
E.ortleppi           GAATTTACCGCGTTTGAATGCTTTGAGTGCGTGACTTTTAGTTCCTTCATTGGTTTTTTT
E.multilocularis     GAATTTGCCACGTTTGAATGCTTTGAGTGCGTGGCTTTTGATTCCTTCATTAGTTTTGTT
E.equinus            GAATTTACCGCGTTTGAATGCTTTGAGTGCGTGGCTTTTGATTCCTTCATTGGTTTTGTT
E.vogeli             GAATTTGCCTCGTTTGAATGCTTTAAGTGCGTGGCTTTTGGTACCTTCGTTGGTTTTTTT
E.oligarthrus        GAATTTGCCTCGTTTAAATGCTTTAAGTGCGTGGCTTTTGGTTCCTTCGTTGATTTTATT
E.felidis            GAATTTACCGCGTTTGAATGCTTTGAGTGCTTGACTTTTGGTTCCTTCGTTGGTTTTGTT
E.granulosus         GAATTTACCGCGTTTGAATGCTTTGAGTGCTTGACTTTTGATTCCTTCGTTGGTTTTTTT
                     ******.** *****.********.***** **.*****..* *****.**..**** **
```

R'-Es

```
                          5′-TGAGTATTAGTGCTGGTTTTGATG-3′
E.shiquicus          TCATATTTGTTTGAGTATTAGTGCTGGTTTTGATGTGTTTGGATTTTATGGGTTATTGTT
E.canadensis G7      TCATATTTGTTTGAGGATTAGTTCTAATTTGGATGTTTTTGGGTTTTATGGGTTGTTGTT
E.canadensis G6      TCATATTTGTTTGAGGATTAGTTCTAATTTGGATGTTTTTGGGTTTTATGGGTTGTTGTT
E.canadensis G10     TCATATTTGTTTGAGGATTAGTTCTAATTTGGATGTTTTTGGGTTTTATGGGTTGTTGTT
E.canadensis G8      TCATATTTGTTTGAGGATTAGTGCTAATTTGGATGTTTTTGGGTTTTATGGGTTGTTGTT
E.ortleppi           TCATATTTGTTTGAGTATCAGTGCTAATTTGGATGTTTTTGGGTTTTATGGGTTGTTATT
E.multilocularis     TCATATTTGTTTAAGTATAAGTGGTAATTTTGATGCGTTTGGGTTTTATGGTTTGTTGTT
E.equinus            TCATATTTGTTTAAGGATTAGTGCTAATTTGGATGTGTTTGGGTTTTATGGGTTGTTGTT
E.vogeli             TCATATATGTTTGAGGGTTAGTGCTAATTTGGATGTGTTTGGGTTTTATGGGTTATTGTT
E.oligarthrus        TCATATATGTCTTAGTATTAGTTCTAATTCGGATGTTTTTGGATTTTATGGGTTGTTGTT
E.felidis            TCATATTTGTTTGAGTATTAGTGCTAATTTAGATGCGTTTGGGTTTTATGGATTGTTGTT
E.granulosus         TCATATTTGTTTGAGTATTAGTGCTAATTTTGATGCGTTTGGGTTCTATGGGTTGTTGTT
                     ******:*** * ** .* ***  *..**  ****  *****.** ***** **.**.**
```
